# Supplementary material for: Learning curve and surgical outcome of robotic assisted colorectal surgery with ERAS program
Source: Sci Rep. 2022 Nov 29;12:20566. doi: 10.1038/s41598-022-24665-w (PMC9709162; doi:10.1038/s41598-022-24665-w)
Supplement: Supplementary file 1 — Supplementary Information 1. [file 41598_2022_24665_MOESM1_ESM.zip › LSC Raw data-Ñ[▒K/144/H_ñw╝╨░O▒Kñσ.pdf]

正式摘要(電子簽章：000000230322996)		
W52 / W52 ☐英文標題(注意:無英文姓名-請補病人基本檔!)	☐【讀取】憑證	

臺中榮民總醫院

出院病歷摘要

轉入號： 轉出號：

[redacted]

(8)流水編號	
(9)入院日期	110/11/14 (2021) 直腸外科 W52 - 122
(10)轉科日期	
(11)出院日期	110/11/20 (2021) 住院天數計 6 日

診斷

- 入院：1. Locally advanced S-colon tumor with liver metastasis and peritoneal seeding cT3N2aM1b, stage IV, with partiel obstruction
- 出院：1. Locally advanced S-colon tumor with liver metastasis and peritoneal seeding cT3N2aM1b, stage IV, with partiel obstruction
  - s/p Laparoscopic AR on 2021/11/15

主訴

- hiccup, epigastralgia and constipation for 2 months

病史

- This 66 y/o female with hypertension under irregular medication control was admitted to hiccup, epigastralgia and constipation for 2 months.
  According to the patient, she suffered from hiccup, gaseous and epigastralgia for 1 month. Accompanied symptoms were small caliber stool and constipation recently. She denied N/V, body weight loss, bloody stool and tenesmus. Thus, she came to Kuang tein hospital for help. CT on 110/10/26 revealed suspect sigmoid colon cancer with non-regional LN, liver, lung metastasis, at least T3N2aM1b and small nodule over right aspect of pelvic cavity, cannot r/o peritoneal metastasis. UGI endoscopy revealed negative finding. LGI endoscopic biopsy proved sigmoid colon adenocarcinoma, moderately differentiated. Chemotherapy with surgical resection was recommended. Then, she came to dr. 蔣 OPD for second opinion. At OPD, lab data showed elevated CA199(23486), CEA(79.39) and impaired liver function(AST:200,ALT:141,ALP:999,LDH:1902, direct bilirubin:0.37) but normal AFP(5.68). CXR showed some small nodular shadow over both lower lung area. Under the impression of sigmoid colon cancer with non-regional LN, liver, lung metastasis, T3N2aM1b, stage IV, with partial obsruction, the patient was admitted to further treatment.

體檢發現

- Anal inspection:
  Recto digital examination:
  Sigmoidoscopy:
  Anoscopy:
  Abdomen:
  - General appearance :
    - chronic ill looking , conscious clear , without cardiopulmonary distress
    - Vital signs自動帶入date 體溫(BT) 脈搏(P) 呼吸(R) 血壓(BP) 身高(BH) 體重(BW) BMI
      20211114 36.3℃ 102次/分 20次/分 147/89mmHg 161.2cm 57.4kg 22.1
    - Vital signs: B.P. 147/89 mmHg , P.R. 102 /min , R.R. 20 /min , B.T. 36.3 ℃
  - HEENT :
    - Eyes: no anemic conjunctiva, no icteric sclera, normal EOM
    - ENT: no otorrhea, no rhinorrhea, no erythematous mucosa, no injected tonsil, no deformity
  - Neck: supple, freely movable, trachea midline,no deformity, no engorged jugular vein , no palpable thyroid or lymph node enlargement
  - Chest:
    - Inspection: Grossly normal, symmetric and fully expansion, no accessory

- muscle used, no operation scar, no spider angiomata, no gynecomastia
    - Palpation: no palpable mass or nodule, no subcutaneous emphysema, no tenderness
    - Ausculation: clear breathing sound
- Heart:
    - Palpation: no thrill, no heave
    - Percussion: normal heart size
    - Ausculation: regular heart beat, normal S1, S2, no gallop, no murmur
- Extremities: No deformity, full ranged of motion without limitation, no clubbing finger or nail cyanosis, no pitting edema, no ulcer or pigmentation, no obvious skin lesion, no varicose vein, no bone, joint or muscle tenderness

CRS CONDITION

- Karnofsky performance status: (100) Normal, no complaints, no evidence of disease
- PE of Abdomen:
- __1. Inspection: no op scar
- __2. Ausculation: not performed
- __3. Palapation: epigastric pain
- __4. Percussion: not performed
- Digital Rectal Examination: not performed
- CEA: 79.39 ng/ml
- Imaging Studies:
- Abnormal Lab data: CA199(23486) and impaired liver function (AST:200,ALT:141,ALP:999,LDH:1902, direct bilirubin:0.37)
- Colonoscope report: LGI endoscopic biopsy proved sigmoid colon adenocarcinoma, moderately differentiated
- Sono , CT of abdomen and chest Films:suspect sigmoid colon cancer with non-regional LN, liver, lung metastasis, at least T3N2aM1b and small nodule over right aspect of pelvic cavity, cannot r/o peritoneal metastasis
- Figures:
    1. colon & rectum

手術日期及方法

- 手術日期及方法20211115 1. Laparoscopic anterior resection
  2. Intraoperative colonoscopy : (-)
  3. High ligation (+) : At IMA root
  4. EndoGIA (60-4.1) X1 were applied for the initial resection. Then another staple of EndoGIA (60-4.1) was applied over the upper rectal stump under the concern of the poor blood supply condition. The anastomosis was completed via firing EEA (29mm).
  # In short, totally 2 staples of EndoGIA was applied.
  5. Proximal cut end: 5 cm, distal cut end: >5 cm
  6. No drainage tube was placed into the abdominal cavity
  7. The ADHESION BARRIER FILM was then placed below the central extended wound.
  8. The colon was not taken down from the splenic flexure site

---

住院治療經過

- After admission, she received Laparoscopic AR on 2021/11/15. She start try Bland I diet on 11/16, and start try bland II diet on 11/17. We shift abx to Unasyn since 11/17. Try Bland III on 11/18 and off PCA since 11/19. Fever had been noticed and subside after Naproxen use. She tolerate wound pain well and no fever noticed again. Thus, she was discharged on 2021/11/20.

合併症

一般檢查

- 累積報告 :

DATE	TIME	ALB	TP	BIL.T	BIL.D	ALKP	AST	ALT	LDH	AMY	LIPASE	NH3	Ferritin	UA
1101115	2045	2.8		1.0	0.5		76	96						
1101118	0854	2.7		0.8	0.4		63	52						

DATE	TIME	WBC	RBC	HGB	HCT	MCV	PLT	BLST	NEUT	BAND	SEG	LYM	MON	EOS	BAS
1101115	2045	12100	3.58	10.6	33.2	92.7	374		78.1			14.5	4.2	2.9	0.3
1101118	0857	12840	3.64	10.6	33.7	92.6	367		83.2			9.4	3.6	3.6	0.2

DATE	TIME	NA	K	CL	BUN	CREAT	eGFR	CA	Mg	P	CRP	ESR	PCT	D-Dimer	LACTATE

-

1101115	2045	136	4.2		6	0.57	112.79								
1101118	0854	135	4.5		5	0.57	112.79	7.8							

特殊檢查

病理報告

其它

出院時情況

- 3.改門診治療

出院指示

-

藥名	劑量	劑量單位	用法	頻次	數量	數量單位	現況	天數	單位
Unasyn TAB 375mg	1	TAB	PO	Q12H	10	TAB	TH	5	天
Acetylcysteine tab	1	TAB	PO	QN	11	TAB	TH	11	天
Ultracet tab	1	TAB	PO	Q6H	44	TAB	TH	11	天
Hirudoid Cream 40gm	1		TOP	BID	2		TH	1	瓶
(□)Takepron OD tab 30mg	1	TAB	PO	QDAC	11	TAB	TH	11	天

出院計劃

- 門診日期
  - date on 2021/11/30

主治醫師 2062F 蔣鋒帆 / 住院醫師 2339C 蔡濬樟

正式摘要**(電子簽章：000000230322996)**

W52 / W52

doctorRank:B
